# A Blood Supply Pathophysiological Microcirculatory Mechanism for Long COVID

**DOI:** 10.3390/life14091076

**Published:** 2024-08-28

**Authors:** Aristotle G. Koutsiaris

**Affiliations:** Medical Informatics and Biomedical Imaging (MIBI) Laboratory, Faculty of Medicine, School of Health Sciences, University of Thessaly, Biopolis Campus, 41500 Larissa, Greece; ariskout@otenet.gr

**Keywords:** long COVID, pathophysiology, microcirculation, mechanism, blood supply, case–control studies, microvascular loss, microthrombosis, hemodynamics, diffusion

## Abstract

Background: The term “Long COVID” is commonly used to describe persisting symptoms after acute COVID-19. Until now, proposed mechanisms for the explanation of Long COVID have not related quantitative measurements to basic laws. In this work, a common framework for the Long COVID pathophysiological mechanism is presented, based on the blood supply deprivation and the flow diffusion equation. Methods: Case–control studies with statistically significant differences between cases (post-COVID patients) and controls, from multiple tissues and geographical areas, were gathered and tabulated. Microvascular loss (ML) was quantified by vessel density reduction (VDR), foveal avascular zone enlargement (FAZE), capillary density reduction (CDR), and percentage of perfused vessel reduction (PPVR). Both ML and hemodynamic decrease (HD) were incorporated in the tissue blood supply reduction (SR) estimation. Results: ML data were found from 763 post-COVID patients with an average VDR, FAZE, CDR, and PPVR of 16%, 31%, 14%, and 21%, respectively. The average HD from 72 post-COVID patients was 37%. The estimated SR for multiple tissues with data from 634 post-COVID patients reached a sizeable 47%. This large SR creates conditions of lower mass diffusion rates, hypoxia, and undernutrition, which at a multi-tissue level, for a long time, can explain the wide variety of the Long COVID symptoms. Conclusions: Disruption of peripheral tissue blood supply by the contribution of both ML and HD is proposed here to be the principal cause of the mechanism leading to Long COVID symptoms.

## 1. Introduction

When the World Health Organization (WHO) declared, on 5 May 2023, the end of the emergency for COVID-19, there was still an increase in confirmed COVID-19 deaths at several places in African, American, European, Southeastern Asia, and Western Pacific regions [1]. Currently, over 7 million deaths and over 775 million confirmed cases have been reported globally [2]. It should be noted that these numbers are underestimations since many countries have stopped or changed the frequency of reporting [2].

Some people cannot fully recover after the COVID-19 disease, presenting long-term symptoms which are usually named “Long-COVID-19”, “Long COVID syndrome”, “post-COVID-19 condition”, or “post-acute COVID-19 syndrome” [3,4]. In short, the term “Long COVID” is commonly used to describe signs and symptoms that persist after acute COVID-19, and this term is going to be used here.

COVID-19 disease affects a multitude of organs and systems, and Long COVID, as a post-infection condition, relates to various systems such as the respiratory, cardiovascular, and nervous systems, underpinning the need for elucidating the exact pathophysiological mechanism [5]. COVID-19 not only significantly reduces axial blood microvessel velocity but also has the devastating effect of causing extensive microthrombosis, as was shown by recent quantitative work on post-COVID patients [6].

Soon after, a normative velocity model in the exchange microvessels was proposed and tested successfully as a disease discriminator on post-COVID conjunctival data [7]. However, microvascular loss (ML), due to microthrombosis, was not taken into account in the normative velocity model because only functional microvessels (with blood flow) were included in the velocity measurements. In reality, the tissue blood supply was even lower due to micro-occlusion. In addition, data in those papers [6,7] were taken only from the conjunctival tissue and a limited geographical region.

In this work, Long COVID symptoms are described first, according to the available data. Then, since most of these symptoms can be attributed to microvascular deprivation of proper blood supply, the concept of microvascular blood supply (S) per flat tissue area is defined. With S, the contribution of both the microvascular hemodynamic decrease (HD) and the ML is taken into account in post-COVID patients. Tissue blood supply reduction (SR) for a long time is the cause of persistent lower mass diffusion rates and therefore multiple tissue hypoxia and undernutrition which can explain most of the Long COVID symptoms. Evidence in support of this mechanism is presented from previously published case–control microcirculatory data from multiple tissues and geographical areas around the world.

## 2. Background on Long COVID and Symptoms

Paul Garner was the first to describe in a blog of the British Medical Journal (5 May 2020) his severe post-COVID symptoms, ongoing 7 weeks after infection [8]. The term “Long COVID” was first used two weeks later by Elisa Perego (20 May 2020), to describe the cyclical and multiphasic long-term COVID illness [8]. Carfi et al. [9] were the first to report symptoms from a cohort of 143 patients that persisted for 36 days (on average) after hospital discharge, and 87% of the patients suffered persistence of at least one symptom, especially fatigue (53%), dyspnea (43%), joint pain (27%), and chest pain (22%). In a multistate survey of 270 COVID-19 adults with milder outpatient illness [10], only 65% had returned to their usual health state when interviewed 2–3 weeks after positive testing. In comparison, more than 90% of influenza outpatients recover within 2 weeks after positive testing [10]. Puntmann et al. [11] were the first to report a cardiovascular magnetic resonance imaging cohort study of 100 patients who recently recovered from COVID-19, and in their work, cardiac involvement and ongoing myocardial inflammation were demonstrated in 78% and 60% of the patients, respectively. In addition, they found a statistically significant discrimination between controls and post-COVID cases using C-reactive protein (CRP), a blood biomarker that positively correlates with the incidence of thrombosis.

According to a 6-month retrospective cohort study of 273,618 survivors of COVID-19 [12], 57% had one or more Long COVID features recorded during the whole 6-month period. The most commonly reported symptoms by order of percentage were anxiety/depression (23%), abnormal breathing (19%), abdominal symptoms (16%), fatigue/malaise (13%), chest/throat pain (13%), other pain (12%), headache (9%), cognitive symptoms (8%), and myalgia (3%). Huang et al. [13] reported that 76% of 1733 COVID-19 patients discharged from the hospital had at least one symptom 6 months after the acute infection. There were similar reports for milder cases of COVID-19 without hospitalization [3], where 61% of patients older than 46 years had persistent symptoms at a 6-month follow-up.

Rezel-Potts et al. [14] conducted a large case–control study with 428,650 COVID-19 patients with a maximum follow-up time of 52 weeks after diagnosis and found that cardiovascular disease was increased in the early COVID-19 phase (first 4 weeks) with principal manifestations of pulmonary embolism, atrial arrhythmias, and venous thromboses (5-fold increase). The 5-fold increase in venous thromboses aligns closely with the 6-fold increase in conjunctival microthrombosis reported in COVID-19 survivors soon after hospital discharge [6]. Katsoularis et al. [15] conducted a nationwide matched cohort study and found that COVID-19 is an independent risk factor for deep vein thrombosis, pulmonary embolism, and bleeding and that the risk of these outcomes is increased for three, six, and two months after COVID-19, respectively.

Other researchers have reported fatigue as a major manifestation of Long COVID with a high prevalence (60–70%) [16,17,18,19]. Poor sleep quality is another common manifestation of Long COVID [16,19,20], and there were demonstrations of chronic pain either as a newly developed condition or a worsening of preexisting chronic pain [21]. The important neurological and psychiatric symptoms observed during Long COVID greatly puzzle scientists worldwide [22]. In addition, a recent retrospective study [16] of 287 patients with Long COVID symptoms found that fatigue, sleepiness, and sleep quality were SARS-CoV-2 variant-independent. This supports the view of a common Long COVID pathophysiological mechanism for all virus variants, which is presented in the next section.

## 3. Materials and Methods

### 3.1. The Normative Range (NR) Diagram

**Figure 1:**
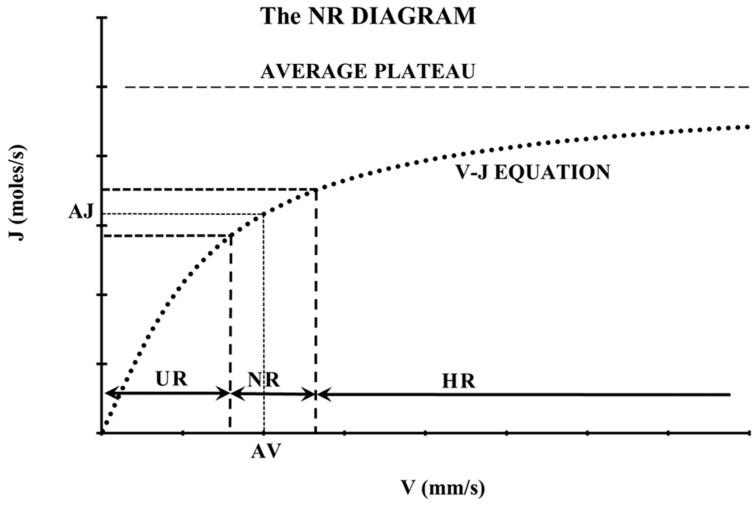
The velocity–diffusion (V-J) equation (black dot curve) and the normative range (NR) diagram [7]. After measuring axial blood velocities at many microvessels with the same diameter, from the same tissue of many healthy persons (control group), an average axial velocity (AV) and a normative range (NR) can be determined statistically. AJ is the average mass diffusion rate corresponding to AV. An “underemic range” (UR) and “hyperemic range” (HR) can be defined as corresponding to average velocities (case group) on the left and the right of the NR, respectively.

The recently introduced velocity–diffusion (V-J) equation [23] is based on the well-known flow–diffusion (Q-J) equation [24], which is one of the fundamental equations of vascular physiology. The V-J equation describes the relation of the axial blood microvessel velocity (V) with the mass diffusion rate (J) of each blood solute through the walls of a microvessel (Figure 1).

After measuring axial velocities at many microvessels with the same diameter, from the same tissue of many healthy subjects (control group), an average axial velocity (AV) can be estimated statistically corresponding to an average mass diffusion rate (AJ). Since the AV is determined statistically, a normative range (NR) of the AV can be defined for microvessels of this diameter, corresponding to normal resting conditions, in a normative range (NR) diagram (Figure 1). The UR (underemic range) is a range of average axial velocities corresponding to underemic conditions on the left of the NR, and the HR (hyperemic range) is the range of average axial velocities corresponding to hyperemic conditions on the right of the NR. Underemic conditions are indicative of hypoxia, malnutrition, and disease. The NR diagram is the backbone of the normative microvascular velocity model and can be extended to many diameters [7].

### 3.2. Hemodynamic Decrease (HD)

In case–control hemodynamic studies, when a statistically significant reduction in the average blood velocity is detected in the case (post-COVID) group, it is direct proof that the average axial velocity (AV) of the case group is shifted to the left, in the underemic range (Figure 1). To quantify this shift, “hemodynamic decrease” (HD) is defined here as follows:

(1) $$HD=\frac{{AV}_{CONTROL}–{AV}_{COVID}}{{AV}_{CONTROL}}100\;(\%)$$

where AV_CONTROL_ and AV_COVID_ are the average axial blood velocities in the control group and the post-COVID group, respectively. HD ranges between 0% and 100% (for AV_CONTROL_ > AV_COVID_). HD can be quantified with various techniques such as Conjunctival Video Capillaroscopy (CVC) [25,26], Laser Doppler Flowmetry (LDF) [27], and special magnetic resonance imaging (MRI) [28].

### 3.3. Microvascular Loss (ML)

“Microvascular loss” (ML) is defined generally as a deprivation of microvessels in a given tissue. ML can be detected in various tissues with various techniques such as Optical Coherence Tomography Angiography (OCTA), CVC, sublingual video capillaroscopy (SVC), and nailfold video capillaroscopy (NVC).

In the subsections below, the well-known indices of vessel density (VD), foveal avascular zone (FAZ), capillary density (CD), and percentage of perfused vessels (PPV) are utilized to make quantitative estimates of ML from case–control studies.

#### 3.3.1. Vessel Density Reduction (VDR)

Vessel density (VD) is defined as the total functional microvessel area per unit area in the region of measurement and is measured in percent (%). Functional microvessels are considered those with blood flow since most techniques require blood flow to detect a microvessel [29]. VD reduction (VDR) is defined as follows:

(2) $$VDR=\frac{{VD}_{CONTROL}-{VD}_{COVID}}{{VD}_{CONTROL}}100\;(\%)$$

where VD_CONTROL_ and VD_COVID_ are the averages in the control and the post-COVID group, respectively. For VD_CONTROL_ > VD_COVID_, VDR ranges between 0% and 100%.

#### 3.3.2. Foveal Avascular Zone Enlargement (FAZE)

The foveal avascular zone (FAZ) is an approximately circular area without any blood vessels with a diameter of about 0.5 mm, located at the center of the fovea in the eye fundus. The FAZ, however, receives proper supply due to the special tissue architecture of that retinal section (foveal pit or foveola about 0.35 mm in diameter). FAZ enlargement (FAZE) is defined as follows:

(3) $$FAZE=\frac{{FAZ}_{COVID}-{FAZ}_{CONTROL}}{{FAZ}_{CONTROL}}100\;(\%)$$

where FAZ_CONTROL_ and FAZ_COVID_ are the averages in the control and the post-COVID group, respectively. FAZE can be considered a kind of microvascular loss in the most critical area of vision.

#### 3.3.3. Capillary Density Reduction (CDR)

Capillary density (CD) is defined as the total capillary number per linear millimeter and is measured by nailfold video capillaroscopy (NVC). Capillary density reduction (CDR) is defined as follows:

(4) $$CDR=\frac{{CD}_{CONTROL}-{CD}_{COVID}}{{CD}_{CONTROL}}100\;(\%)$$

where CD_CONTROL_ and CD_COVID_ are the averages in the control and the post-COVID group, respectively. CDR ranges between 0% and 100 (for CD_CONTROL_ > CD_COVID_).

#### 3.3.4. Percentage of Perfused Vessel Reduction (PPVR)

The percentage of perfused vessels (PPV) is defined as the percentage of the number of perfused (functional) vessels over the total number of vessels, at a given microvascular area (field of view). The PPV is an index inversely related to the microvascular damage (thrombotic micro-occlusion) sustained by a tissue. The percentage of perfused vessels reduction (PPVR) is defined as follows:

(5) $$PPVR=\frac{{PPV}_{CONTROL}-{PPV}_{COVID}}{{PPV}_{CONTROL}}100\;(\%)$$

where PPV_CONTROL_ and PPV_COVID_ are the averages in the control and the post-COVID group, respectively. PPVR ranges between 0% and 100% (for PPV_CONTROL_ > PPV_COVID_).

### 3.4. Blood Supply Reduction (SR)

In case–control microvascular studies, when a statistically significant ML is detected in the tissue of the case group, it is evident that there is an oxygen and nutrient undersupply in that tissue area. The current difficulty with the normative velocity model of Figure 1 is that ML is not taken into account. It is as if Figure 1 has many “zero points” (V = 0, J = 0) which are not measured. However, the higher the average zero points, the higher the tissue undersupply.

To overcome this difficulty, the blood supply (S) per flat tissue area is defined. For a given flat tissue surface area (field of view) of a subject, the blood supply S is the sum of all the blood volume flows (Q_1_ + Q_2_ + … + Qn) of the n functional exchange microvessels. This can be written as S = n Q, where Q is the average blood volume flow of the n microvessels. This can be written as S = n b V, where b is a parameter for the average cross-sectional area (m^2^) of the n microvessels and V is the average axial blood velocity (m/s) of the n microvessels. In this way, changes in the average number of functional microvessels can be incorporated into the S calculations.

In a case–control study with post-COVID patients, the average blood supply per flat tissue area for the control group can be expressed as S_CONTROL_ = n_CONTROL_•b_CONTROL_•V_CONTROL_, where S_CONTROL_, n_CONTROL_, b_CONTROL_, and V_CONTROL_ are the control group averages for S, n, b, and V, respectively. In the same manner, for the post-COVID group, S_COVID_ = n_COVID_•b_COVID_•V_COVID_.

In a situation where an average reduction in alpha (α) percent was observed in the number of microvessels in the post-COVID group, then n_COVID_ could be expressed as [(100 − α)/100]n_CONTROL_. If on surplus an average hemodynamic reduction in HD percent was observed in the remaining functional microvessels, then V_COVID_ could be expressed as [(100 − HD)/100]V_CONTROL_. Therefore, the average post-COVID group blood supply is written as follows:

(6) $$S_{COVID}=\left[\frac{\left(100-\alpha \right)}{100}n_{CONTROL}\right]•b_{COVID}•\left[\frac{\left(100-HD\right)}{100}V_{CONTROL}\right]$$

With the condition that the average cross-sectional area of the remaining functional microvessels in the post-COVID group is the same as that in the control group (b_CONTROL_ = b_COVID_), Equation (6) is written as follows:

(7) $$S_{COVID}=\frac{\left(100-\alpha \right)(100-HD)}{10,000}S_{CONTROL}$$

Finally, the blood supply reduction (SR) in percent is defined as follows:

(8) $$SR=\frac{S_{CONTROL}-S_{COVID}}{S_{CONTROL}}100\;(\%)$$

After inserting Equation (7) into Equation (8), we obtain the following:

(9) $$SR=100-\frac{\left(100-\alpha \right)\left(100-HD\right)}{100}\;(\%)$$

Using Equation (9), the total supply reduction SR from both the microvascular hemodynamic decrease (HD) and the microvascular loss (α) is taken into account. Of the four microvascular loss quantitative measures (VDR, FAZE, CDR, and PPVR), only two were defined on a flat tissue area: VDR and PPVR. Here, it is considered that α can be approximated by VDR or PPVR depending on the available data.

### 3.5. The Proposed Pathophysiological Microcirculatory Mechanism for Long COVID

**Figure 2:**
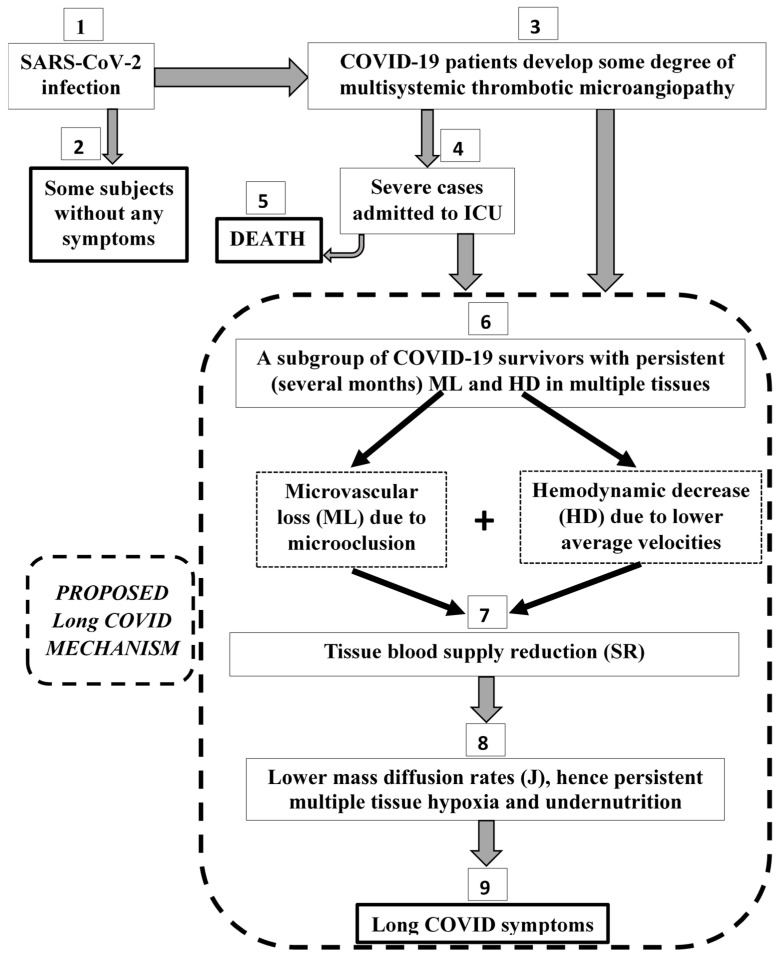
The steps and possible outcomes of the COVID-19 disease progress are shown in numbered rectangular boxes. Boxes with end states are shown in solid bold black lines. The common framework for the proposed Long COVID mechanism is represented by boxes 6, 7, 8, and 9, which are inside the dashed black line. ICU: Intensive Care Unit, ML: microvascular loss, HD: hemodynamic decrease, NR: normative range, J: mass diffusion rate (moles/s).

The steps and possible outcomes of the COVID-19 disease progress are shown in numbered rectangular boxes (Figure 2, boxes 1–9). The common framework for the proposed Long COVID mechanism is represented schematically by boxes 6, 7, 8, and 9, which are included in a dashed black line (Figure 2). Box 6 represents the subgroup of COVID-19 survivors with microvascular loss (ML) and hemodynamical decrease (HD) for several months after testing negative. Box 7 shows that both ML and HD lead to tissue blood supply reduction (SR). The significant SR has consequently much lower diffusion rates (J) hence, multiple tissue hypoxia and undernutrition (box 8), which can be considered the cause of the Long COVID symptoms (box 9).

### 3.6. Selection of Case–Control Studies

The article screening included publications until 30 April 2024, from PubMed. In the PubMed Advanced Search Builder, the search term combination (“covid 19” [Title/Abstract] AND (“microvascular” [Title/Abstract] OR “microcirculation” [Title/Abstract] OR “velocity” [Title/Abstract])) gave 1340 results. From those results, the selection procedure included only case–control original articles with patients in the recovery phase of the COVID-19 disease (after hospitalization or after having tested negative). These patients are called “post-COVID cases” in short. In addition, the selection procedure included only those case–control papers reporting results of ML measures (VDR, FAZE, CDR, or PPVR) or hemodynamic decrease (HD). In vitro studies, simulations, and review articles were excluded.

Seventeen papers with ML results were identified [30,31,32,33,34,35,36,37,38,39,40,41,42,43,44,45,46] with VDR results from the retina [30,31,32,33,34,35,36,37,40,41], the choroid [43], and the sublingual tissue [44], with FAZE results from the retina [31,32,33,37,38,39,41], and with CDR results from the nailfold [45,46]. One paper was found with PPVR results from the conjunctiva [6].

According to a recent meta-analysis by Kazantzis et al. [47] that investigated retinal microcirculation changes in patients recovered from COVID-19 infection, in comparison to healthy controls, from 12 eligible research studies, the following was observed: (1) parafoveal vessel density in the deep capillary plexus was significantly lower in 365 post-COVID patients compared to healthy controls (Higgins I^2^ < 25%), and (2) the whole image FAZ area was significantly larger in 370 post-COVID patients compared to healthy controls (I^2^ < 25%). It is noted that all available studies (with significant and not significant differences) were included in this meta-analysis [47]. The results of this meta-analysis [47] proved that there is a statistically significant VDR and FAZE in the human eye, and this was taken into account as a standard in this work for all tissues, and only studies with statistically significant ML metrics (VDR, FAZE, CDR, and PPVR) are in the selected seventeen papers [30,31,32,33,34,35,36,37,38,39,40,41,42,43,44,45,46].

Four case–control papers with microvascular hemodynamics quantification (HD) were identified [6,27,28,44]. One of them [44] was excluded from hemodynamic analysis because it did not report results in the diametric microvascular range between 7 and 10 microns. In addition, the cases group was divided into severe and mild cases, and the measurement technique required tissue contact, which may affect microvascular hemodynamics.

### 3.7. Statistical Analysis

The Microsoft Office EXCEL 2016 software (professional edition) was used for the estimation of median, mean, and standard error of the mean (SEM). The statistical estimations were performed on different tissues since HD and ML metrics were all defined in relative units (percentages) to the control group reference.

## 4. Results

### 4.1. Hemodynamic Decrease (HD) Case–Control Studies [Table 1]

In Table 1, case–control data are shown from 72 post-COVID patients, where a statistically significant HD was measured in three different tissue types (conjunctiva, skin, and brain), from three distant countries: Greece, Russia, and China. The reported HD ranged between 29% (*p* < 0.05) and 45% (*p* < 0.001) with a mean value of 37% and a standard error of the mean (SEM) of 8%.

**Table 1:** Post-COVID case–control studies with statistically significant hemodynamic decrease (HD).

TISSUE	STUDY	VASCULAR BED/METHOD	HD (%)	N
Conjunctiva	Koutsiaris et al. [6]	exchange microvessels/CVC	45	17
Skin	Zharkikh et al. [27]	wrist and shin microvessels/LDF	29	23
Brain	Qin et al. [28]	gray matter cortex, subcortical nuclei/MRI	-	32
MEDIAN			37	-
MEAN ± SEM			37 ± 8	-
RANGE			16	-
TOTAL				72

Data refer to a post-COVID period of 0 to 6 months. The HD of 45% [6] is the average among 3 different microvessel classes (capillaries, and postcapillary venules of size 1 and 2). The HD of 29% [27] is the average between the wrists and shins. N: number of post-COVID patients, CVC: Conjunctival Video Capillaroscopy, LDF: Laser Doppler Flowmetry, MRI: magnetic resonance imaging, SEM: standard error of the mean.

In summary, regarding the conjunctival microvascular bed, Koutsiaris et al. [6] reported for the early post-COVID patients a highly statistically significant (*p* < 0.001) reduction in V (39%, 49%, and 47%, for capillaries, postcapillary venules of size 1, and postcapillary venules of size 2, respectively) in comparison to the control group. Their average of 45% is shown in Table 1. The measurements were in absolute units of velocity from the capillaries and postcapillary venules of the microvascular network (exchange microvessels). Regarding the wrist and shin skin, Zharkikh et al. [27] reported impressive reductions of 24% and 34% for nutritive microvessel blood flow at the wrists and shins of post-COVID subjects, respectively. Their average of 29% is shown in Table 1. Their statistically significant results were measured by Laser Doppler Flowmetry (LDF) and reinforced the opinion of microvascular dysfunction for a long period (1 to 6 months) after the recovery from COVID-19. Regarding the brain, Qin et al. [28] found statistically significant lower blood flow in the brain cortex and subcortical nuclei of 32 post-COVID patients (severe cases) 3 months after discharge. Their indirect measurements were based on a kinetic model applied to pseudo-continuous arterial spin labeling images.

### 4.2. Microvascular Loss (ML) Case–Control Studies [Table 2, Table 3, Table 4 and Table 5]

Post-COVID case–control data [30,31,32,33,34,35,36,37,38,39,40,41,42,43,44,45,46] of statistically significant microvascular loss (ML) are available zero to six months after hospital exit or after testing negative. These data come from [A] the retina (measured by OCTA, Table 2 and Table 3), [B] the choroid (measured by OCTA, Table 2), [C] the sublingual tissue (measured by SVC, Table 2), [D] the nailfold (measured by NVC, Table 4), and [E] the conjunctiva (measured by CVC, Table 5).

**Table 2:** Post-COVID case–control studies with statistically significant vessel density reduction (VDR).

TISSUE	STUDY	VASCULAR BED	VDR (%)	N
Retina	Savastano et al. [30]	RPCP	3	80
	Gonzalez-Zamora et al. [31]	foveal SCP	48	25
		foveal DCP	33	
	Bilbao-Malavé et al. [32]	foveal SCP	51	17
	Abrishami et al. [33]	foveal DCP	13	31
	Guemes-Villahoz et al. [34]	SCP and DCP	7	66
	Hazar et al. [35]	superior sector DCP	2	50
	Cennamo et al. [36]	whole image RPCP	8	40
	Erogul et al. [37]	whole image SCP	3	32
	Kalaw et al. [40]	3 inner retinal layers	8	7
	Urfalioğlu et al. [41]	DCP	3	72
	El-Hadad et al. [42]	Deep macular plexus	11	50
		RPCP	6	
Choroid	Üçer and Cevher [43]	choroidal microvessels	6	65
Sublingual	Osiaevi et al. [44]	sublingual microvessels	41	27
MEDIAN			8	-
MEAN ± SEM			16 ± 5	-
RANGE			49	-
TOTAL				562

Data refer to a post-COVID period of 0 to 6 months except for the study of Osiaevi et al. [44] that measured up to 18 months after infection. N: number of post-COVID patients, RPCP: radial peripapillary capillary plexus, SCP: superficial capillary plexus, DCP: deep capillary plexus, SEM: standard error of the mean.

**Table 3:** Post-COVID case–control studies with statistically significant foveal avascular zone enlargement (FAZE) in the human retina.

STUDY	VASCULAR BED	FAZE (%)	N
Gonzalez-Zamora et al. [31]	SCP	55	25
Bilbao-Malave et al. [32]	SCP	65	17
Abrishami et al. [33]	whole image (SCP and DCP)	12	31
Erogul et al. [37]	whole image (SCP and DCP)	11	32
Dipu et al. [38]	SCP	19	35
	DCP	15	
Kal et al. [39]	SCP	30	63
	DCP	51	
Urfalioğlu et al. [41]	DCP FAZ	20	72
MEDIAN		20	-
MEAN ± SEM		31 ± 7	-
RANGE		54	-
TOTAL			275

Data refer to a post-COVID period of 0 to 6 months. N: number of post-COVID patients, SCP: superficial capillary plexus, DCP: deep capillary plexus. SEM: standard error of the mean.

**Table 4:** Post-COVID case–control studies with statistically significant capillary density reduction (CDR) in the human nailfold.

STUDY	VASCULAR BED	CDR (%)	N
Çakmak et al. [45]	finger nailfold capillaries	17	25
Sulli et al. [46]	finger nailfold capillaries	11 *	61
MEDIAN		14	-
MEAN ± SEM		14 ± 3	-
RANGE		6	-
TOTAL			86

Data refer to a post-COVID period of 0 to 10 months except for the study of Sulli et al. [46] that measured patients up to 10 months after hospital discharge. N: number of post-COVID patients. * The average CDR between mild/moderate (34 subjects) and severe (27 subjects) cases.

**Table 5:** Post-COVID case–control studies with statistically significant percentage of perfused vessel reduction (PPVR) in the human conjunctiva.

STUDY	VASCULAR BED	PPVR (%)	N
Koutsiaris et al. [6]	eye conjunctiva	21	17

Data refer to a post-COVID period of 0 to 1 months. N: number of post-COVID patients.

According to these data, there was a statistically significant microvascular loss (ML) observed in 763 post-COVID patients. The results from these data are presented in the subsections below with quantitative estimates of the defined ML measures (Table 2, Table 3, Table 4 and Table 5).

#### 4.2.1. Vessel Density Reduction (VDR) Case–Control Studies [Table 2]

In Table 2, case–control data are shown from 562 post-COVID patients, where a statistically significant VDR was measured in three different tissues: the retina, the choroid, and the sublingual tissue [30,31,32,33,34,35,36,37,40,41,42,43,44]. The reported VDRs ranged between 2 and 51% with a mean value of 16% and a standard error of the mean (SEM) of 5%.

In summary, regarding retinal microcirculation, Savastano et al. [30] were the first to report a significantly lower vessel density in the radial peripapillary capillary plexus (RPCP) of 80 post-COVID patients, using OCTA, a relatively new, noninvasive, noncontact, imaging technique [29] which makes the collection of retinal microcirculatory measurements much easier than before. A series of other studies in the human retina [31,32,33,34,35,36,37,41,42,43,44,45,46,47] have reported a statistically significant VDR, also using OCTA. Regarding the choroidal microcirculation, Üçer and Cevher [43] found a statistically significantly reduced choroidal vascularity index in 65 post-COVID patients in comparison to the control group. Regarding the sublingual microvessels, Osiaevi et al. [44] measured capillary density using a kind of videomicroscopy called sidestream dark field imaging.

#### 4.2.2. FAZ Enlargement (FAZE) Case–Control Studies [Table 3]

In Table 3, case–control data are shown from 275 post-COVID patients [31,32,33,37,38,39,41], where a statistically significant FAZE was measured in the most critical area for proper vision. The reported FAZE ranged between 11 and 65% with a mean (±SEM) value of 31 ± 7%.

#### 4.2.3. Capillary Density Reduction (CDR) Case–Control Studies [Table 4]

In Table 4, case–control data are shown from 86 post-COVID patients [45,46], in whom a statistically significant CDR was measured. The reported CDR ranged between 11 and 17% with a mean (±SEM) equal to 14 ± 4%. In the study of Sulli et al. [46], no drug significantly reversed the CDR in COVID-19 survivors.

#### 4.2.4. Percentage of Perfused Vessel Reduction (PPVR) Case–Control Studies [Table 5]

Regarding the conjunctival microvascular bed, an average PPVR of 21% was reported (Table 5). In summary, Koutsiaris et al. [6] found in the post-COVID group a statistically significant (*p* < 0.001) and sizeable (sixfold) increase in the percentage of occluded vessels in comparison to the control group, due to microthrombosis. The sixfold increase in the percentage of occluded vessels corresponds by definition to a PPVR of 21%. In the same study, the existence of a possibly unknown microvessel coagulation factor was proposed to be triggered by COVID-19.

### 4.3. Blood Supply Reduction (SR) [Table 6]

From HD and PPVR measurements on the same tissue (conjunctiva, Table 1 and Table 5 [6]), the estimated SR is 57% (Table 6). After combining the HD data of Table 1 [conjunctiva, skin, and brain], from 72 patients, with the VDR data from Table 2 [retinal, choroidal, and sublingual tissue], from 562 patients, an SR estimation of 47% is shown in Table 6.

**Table 6:** Blood supply reduction (SR).

TISSUE (DATA SOURCE)	α (%)		HD (%)	SR (%)	N
	PPVR (%)	VDR (%)			
Conjunctiva (Table 1 and Table 5)	21	-	45	57	17
Conjunctiva/Skin/Brain (Table 1)	-	-	37	-	72
Retina/Choroid/Sublingual (Table 2)	-	16	-	-	562
Multiple Tissues (Table 1 and Table 2)	-	16	37	47	634

Data refer to a post-COVID period of 0 to 6 months. α (alpha): average reduction in the number of functional exchange microvessels in the post-COVID group, PPVR: percentage of perfused vessel reduction, VDR: vessel density reduction, HD: hemodynamic decrease, N: number of post-COVID patients.

### 4.4. Results Supporting the Proposed Mechanism

According to available clinical information (Table 1), the axial blood microvessel flow of 72 post-COVID patients was lower than normal with an average hemodynamic decrease (HD) of 37% (Figure 2, box 6). In addition, there was a statistically significant microvascular loss (ML) in 763 post-COVID patients (Table 2, Table 3, Table 4 and Table 5) and specifically, an average vessel density reduction (VDR) of 16% (Table 2, 562 patients), an average foveal avascular zone enlargement (FAZE) of 31% (Table 3, 275 patients), an average capillary density reduction (CDR) of 14% (Table 4, 86 patients), and a percentage of perfused vessel reduction (PPVR) of 21% (Table 5, 17 patients].

The contribution of both hemodynamic decrease (HD) (Table 1) and vessel density reduction VDR (Table 2) is translated to an estimated blood supply reduction SR of 47% in multiple tissues (Table 6), (Figure 2, box 7). This estimation is very close to 57% of the human conjunctiva (Table 6), where HD and PPVR measurements were performed on the same tissue.

The multiple tissue undersupply observed for so long (post-COVID period of 0–6 months, Table 1, Table 2, Table 3, Table 4, Table 5 and Table 6) leads to lower mass diffusion rates (J) according to the normative range diagram (Figure 1). The consequence of the persistent lower J is the undersupply of oxygen (tissue hypoxia), undernutrition, and the under-dispense of waste (Figure 2, box 8).

Hypoxic and undernutrition conditions at a multi-tissue level for a long time can explain the wide variety of the reported Long COVID symptoms (Figure 2, box 9): anxiety, depression, headache, cognitive decline, abnormal breathing, abdominal symptoms, fatigue, sleep disorders, and myalgia (see Section 2). This is especially true for the principal symptoms that derive from the brain, which is the most sensitive organ to oxygen supply: anxiety, depression, headache, cognitive decline, abnormal breathing, and sleep disorders.

## 5. Discussion

Life is sustained by the diffusion of oxygen and nutrients towards the intracellular space. In animals with a circulatory system, this diffusive process takes mainly place in the exchange microvessels where the adequate supply of blood is of uttermost importance. When the normal tissue blood supply is disrupted at the microvascular level, there are serious side effects.

Early in the COVID-19 pandemic, Polak et al. [48] reported the importance of microvascular histological patterns and their persistence throughout the clinical course in their systematic review including 131 lung samples from either antemortem or postmortem COVID-19 patients (Figure 2, boxes 3–5). They identified 76 cases (59%) with microvascular damage (microthrombi) and proposed directed anti-inflammatory, anticoagulant, and/or anticomplement agents. Pretorius et al. [49] detected amyloid microclots in twenty COVID-19-positive blood samples before patient treatment, and Jung et al. [50] reported abdominal microcirculatory disorders (Figure 2, box 3). At the beginning of 2022, coagulopathy in patients hospitalized with COVID-19 was well documented [51], and there were reports of enhanced hypercoagulability [52] and impaired fibrinolysis [53]. Endothelial dysfunction [54] is another characteristic of COVID-19 related to microvascular disease and vascular aging [55].

In this work, a common framework (Figure 2, boxes 6–9) was presented for the pathophysiological mechanism of the Long COVID symptoms reported by COVID-19 patients after hospital exit or after testing negative. After the acute phase (Figure 2, box 3), a multisystemic microvasculopathy persists for several months (Figure 2, box 6), as was presented in the results, for about 800 post-COVID patients (Table 1, Table 2, Table 3, Table 4 and Table 5). This can explain the Long COVID symptoms through the quantitative pathophysiological mechanism of the tissue blood supply reduction (SR) (Figure 2, boxes 7–9).

Regarding post-COVID patients, Patterson et al. [56] in the middle of 2020, reported that SARS-CoV-2 infection is associated with a wide spectrum of neurological syndromes, and D-dimers were markedly elevated in all patient subgroups. In a cohort study of 100 patients who recovered from COVID-19 [11], statistically significant discrimination between controls and post-COVID cases was reported using C-reactive protein (CRP), a blood biomarker that positively correlates with the incidence of thrombosis. Pretorius et al. [57] found microclots that were resistant to fibrinolysis, and a substantial increase in α2-antiplasmin, in blood samples from 11 post-COVID patients at least 2 months after recovery. The presence of amyloid microclots was also reported in blood samples from 80 post-COVID patients [58] (but without controls). Scheim et al. [59] proposed a mechanism of microclot formation with a central role of sialylated glycan attachments between SARS-CoV-2 spike proteins and red blood cells. Kell and Pretorius [60] proposed ischemic injury from fibrin amyloid microclots as the primary factor for the Long COVID condition, and in line with this, Astin et al. [61] proposed lower tissue oxygen availability (chronic hypoxia) as a pathophysiological mechanism of Long COVID. The microclot blocking of capillaries in post-COVID patients was confirmed in vivo by Koutsiaris et al. [6] who also proposed microthrombosis as a possible explanation for Long COVID syndrome and speculated the existence of a possibly unknown coagulation factor.

The propositions mentioned above are well fitted into the pathophysiological context of the present work, with the difference that here, case–control in vivo quantitative data were gathered, from multiple human tissues and multiple geographical places, with statistically significant differences between post-COVID cases and controls (Table 1, Table 2, Table 3, Table 4 and Table 5, Figure 2, box 6).

The major finding of a case–control study [62] with 120 Long COVID individuals 3–4 months after the acute infectious phase was the association of Long COVID with decreased antioxidant defenses as indicated by the lowered total antioxidant capacity of plasma. In addition, a high percentage of the variance in the severity of the Long COVID neuropsychiatric symptoms was explained by the increased C-reactive protein and the ratio of oxidative stress toxicity to antioxidants. These findings are in support of the pathophysiological model resulting in tissue hypoxia in this work (Figure 2, box 8).

In a study with 87 COVID-19 survivors after hospitalization [63], exercise intolerance was reported, and a hypothesis of lung microvascular injury was made, as a pathophysiological mechanism leading to increased dead space as a fraction of tidal volume during exercise in post-COVID-19 patients. This hypothesis fits very well in the context of this work.

Gareau and Barrett [64] proposed a role of the impaired microbiota–gut–brain axis signaling in the development of Long COVID, but this does not explain the extensive microthrombosis. However, a potential link to microthrombosis may exist in the gut–lung axis through the degradation of the intestinal epithelial cell junctional proteins and hence of the biochemical barrier to the microvessels.

Reiss et al. [65] explored possible mechanistic pathways between Long COVID and nervous system inflammation. In their review, it was noted that impaired blood flow in the brain due to viral invasion of the microvascular endothelium may be a neuropathological mechanism of Long COVID which agrees with this work.

In three recent Long COVID reviews [5,66,67], the need for understanding the pathophysiological mechanism of Long COVID was underscored, and various possible cellular and molecular mechanisms were described. Among them, the general concept of endothelial dysfunction damage, microclot formation, persistent microvascular injury, and the impairment of oxygen transfer is in good agreement with this work, which supports it as the most prevalent mechanism.

It should be noted that even though a lot of evidence was presented for the thrombotic cause of microvascular occlusion, the actual cause is not a limitation of this study because the methodology based on quantified hemodynamic decrease (HD) and microvascular loss (ML) is valid irrespective of the background cause.

Limitations of the mathematical model are firstly the definition of a flat tissue area; however, most of the in vivo human microcirculatory data come from flat tissue areas. Secondly, the condition that the average cross-sectional area of the remaining functional microvessels in the post-COVID group is the same as that in the control group (b_CONTROL_ = b_COVID_). Thirdly, another limitation is the approximation of α (Equation (9)) by VDR or PPVR, depending on the available data.

For an indication of when the microvascular status returns to normal, a recent work [68] with retinal evaluations at an average of 15.2 ± 6.9 months post SARS-CoV-2 infection reported no significant differences from controls.

Assuming conservatively that only 10% of the COVID-19-infected people developed Long COVID [69], at least 77.5 million subjects worldwide have shown Long COVID symptoms [2]. With insufficient current Long COVID diagnostic and treatment options [69], more basic and clinical research is needed to understand Long COVID pathophysiological mechanisms.

## 6. Conclusions

In conclusion, a pathophysiological framework for the explanation of Long COVID was presented, based on published quantitative case–control data of reduced peripheral microvascular blood supply from about 800 post-COVID patients. More research data are essential to elucidate the Long COVID mechanism.

## Data Availability

The original contributions presented in the study are included in the article, further inquiries can be directed to the corresponding author.
